# cGAS-STING, an important pathway in cancer immunotherapy

**DOI:** 10.1186/s13045-020-00916-z

**Published:** 2020-06-22

**Authors:** Minlin Jiang, Peixin Chen, Lei Wang, Wei Li, Bin Chen, Yu Liu, Hao Wang, Sha Zhao, Lingyun Ye, Yayi He, Caicun Zhou

**Affiliations:** 1 Department of Medical Oncology, Shanghai Pulmonary Hospital, Tongji University Medical School Cancer Institute, Tongji University School of Medicine, No 507 Zhengmin Road, Shanghai, 200433 People’s Republic of China; 2 Tongji University, No 1239 Siping Road, Shanghai, 200433 People’s Republic of China

**Keywords:** cGAS-STING, Cancer, Combined therapy, Immunotherapy, STING pathway

## Abstract

Cytosolic DNA sensing, the cyclic GMP-AMP synthase-stimulator of interferon genes (cGAS-STING) pathway, is an important novel role in the immune system. Multiple STING agonists were developed for cancer therapy study with great results achieved in pre-clinical work. Recent progress in the mechanical understanding of STING pathway in IFN production and T cell priming, indicates its promising role for cancer immunotherapy. STING agonists co-administrated with other cancer immunotherapies, including cancer vaccines, immune checkpoint inhibitors such as anti-programmed death 1 and cytotoxic T lymphocyte-associated antigen 4 antibodies, and adoptive T cell transfer therapies, would hold a promise of treating medium and advanced cancers. Despite the applications of STING agonists in cancer immunotherapy, lots of obstacles remain for further study. In this review, we mainly examine the biological characters, current applications, challenges, and future directions of cGAS-STING in cancer immunotherapy.

## Background

Cancer is one of the major lethal diseases worldwide, with a high morbidity of 18.1 million estimated new diagnosed cases and mortality of 9.6 million deaths in 2018 reported in the Global cancer statistics [1]. Cancer immunotherapy has made a great breakthrough in oncology, and the discovery of immune checkpoint inhibitors (ICIs) was awarded the 2018 Nobel Prize. Although the application of anti-cytotoxic T lymphocyte antigen 4 (CTLA-4) and anti-programmed death 1 (PD-1) therapies has yielded impressive clinical efficacy, response to these methods only presents in a fraction of patients, and recent evidence has suggested some drug-resistant and lethal cases [2, 3].

**Fig. 1:**
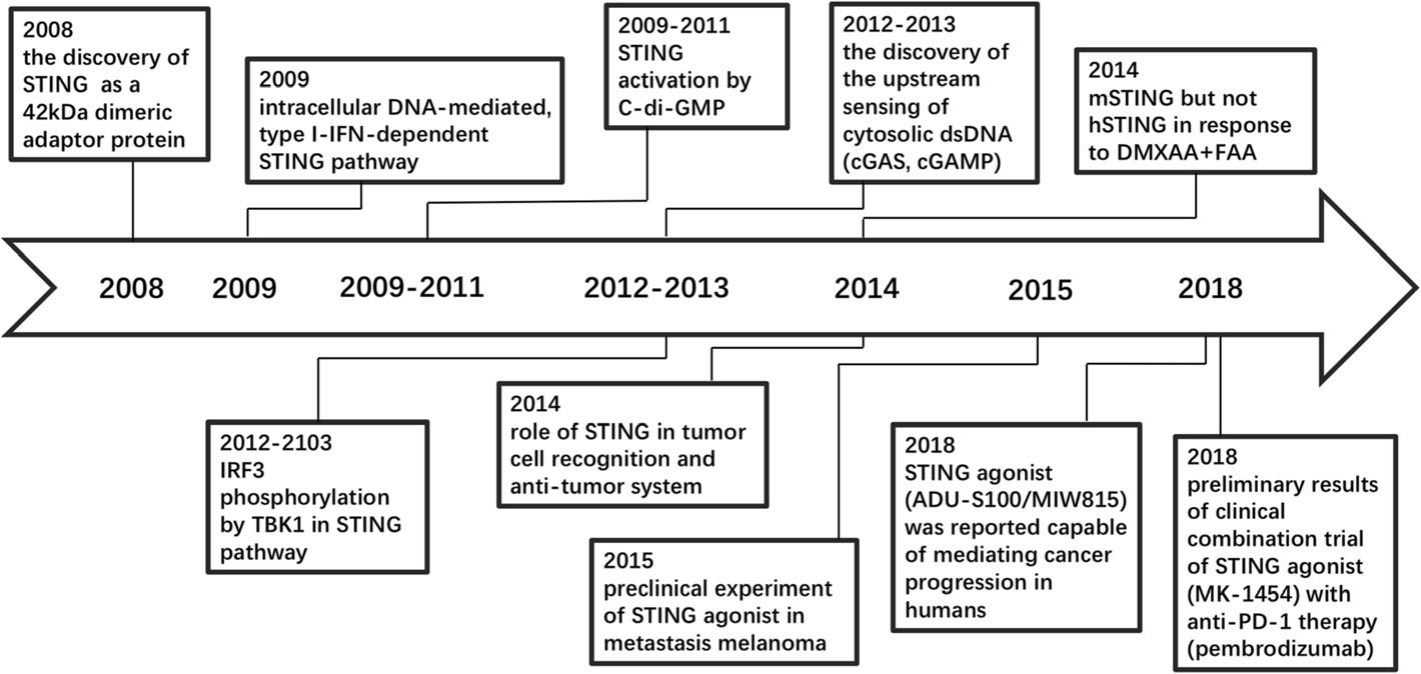
Timeline of the understanding of STING pathway and its role in cancer immunotherapy. Abbreviation: DMXAA: dimethyloxoxanthenyl acetic acid; FAA: Flavone 8-acetic acid; IRF: interferon regulatory factor; TBK1: TANK-binding kinase 1

The stimulator of interferon genes (STING) is a novel player with pleiotropic effects in the field of the immune system. The discovery of STING as a 42-kDa “dimeric adaptor protein” in 2008 quickly expanded the fields of immunology research as well as cancer immunotherapy [4]. The STING-targeted treatment is a novel candidate for anti-tumor immunotherapy and agents such as ADU-S100(MIW815) (NCT02675439), MK-1454(NCT03010176), and E7766(NCT04144140) have been approved for clinical trials to test their capability of mediating cancer progression in human beings. The understanding of the activated STING pathway has made much progress in antitumor responses necessarily via tumor microenvironment (TME) heating-up by interferon (IFN) secretion and lymphocyte infiltration, which is an excitingly promising direction for cancer immunotherapy (Fig. 1). Several excellent reviews showed unique perspectives on the cyclic GMP-AMP synthase (cGAS)-STING pathway, which identify the structural biology of STING protein, its role in the immune system, as well as the regulation and function of it in DNA sensing [5–7]. In this review, we focus on the basis of the application and pharmacological effect of STING agonists as antitumor therapy, the application of STING in antitumor immunotherapy, its limitations, and some feasible suggestions in the use of STING agonists.

## Basis of STING signaling pathway

### cGAS-STING pathway

The cGAS-STING pathway is the central cellular cytosolic double-stranded DNA (dsDNA) sensor, allowing innate immune to respond to infections, inflammation, and cancer [8, 9]. Both intrinsic and extrinsic self-DNA sensing can contribute to its activation. It is clear that the STING pathway is more than just important in pathogen detection, but also plays an important role in the detection of rather the self-DNA released from tumor cells and dying cells [10]. It was also reported that the mitochondrial DNA (mtDNA) instability promoted the escape of mtDNA into the cytosol and activated the antiviral immunity via the cGAS-STING pathway [11].

**Fig. 2:**
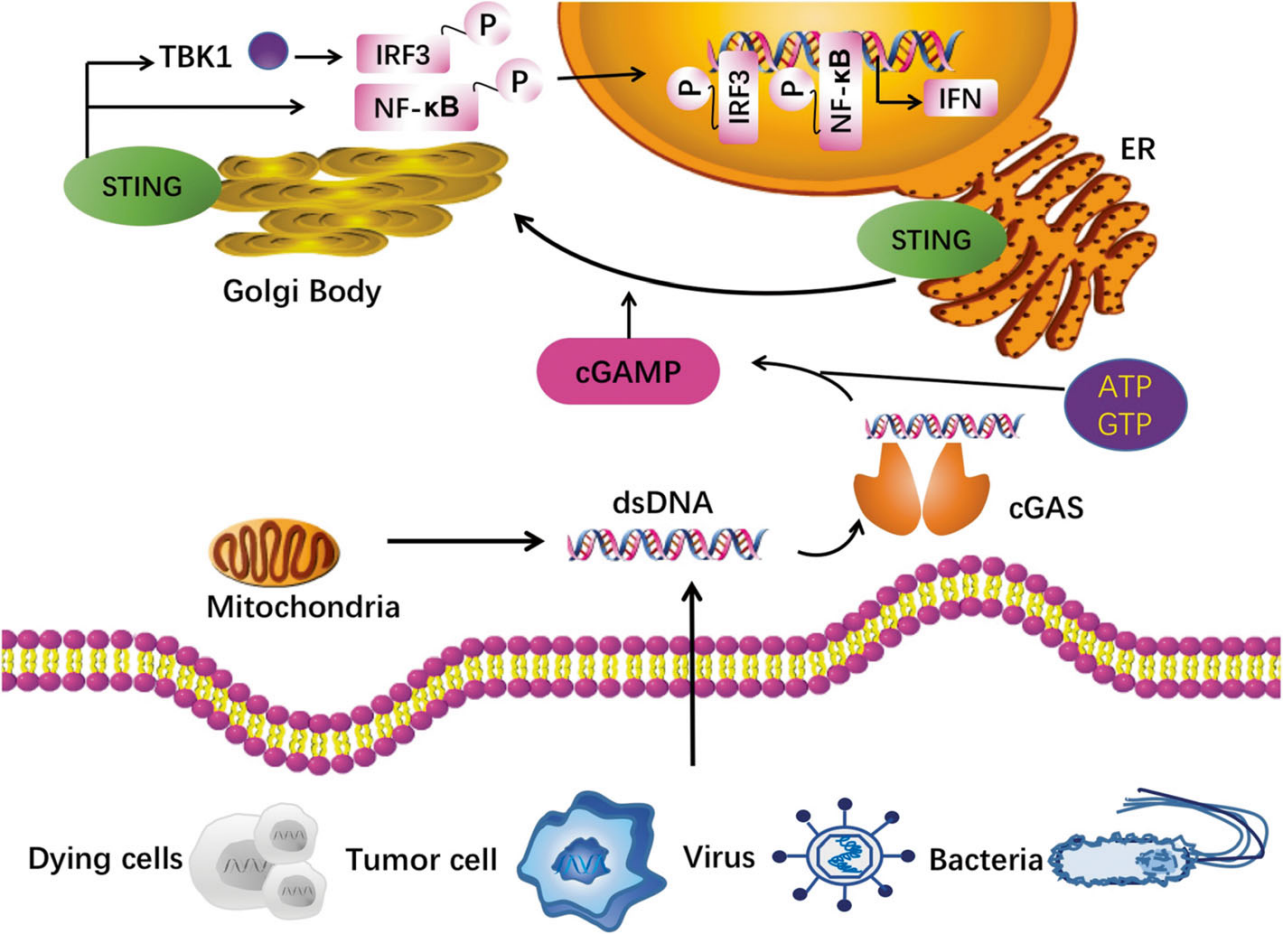
cGAS-STING pathway. Exogenous DNA from dying cell, tumor cell, virus and bacteria, and endogenous DNA leakage from mitochondria, interact with the cytosolic DNA sensor cGAS in a sequence-independent way, promoting a conformational change of cGAS to catalyze the formation of 2′,3′-cyclic GMP-AMP (cGAMP). The cGAS activation as well as cGAMP synthase activate protein STING, in which the STING undergoes endoplasmic reticulum (ER)-to-Golgi trafficking and tetramer formation via a higher-order oligomerization. Palmitoylation of STING in Golgi is proposed for TANK binding kinase 1 (TBK1) as well as interferon regulatory factor 3 (IRF3) recruitment. The STING tetramerization induces recruitment and activation of TBK1 dimers, and TBK1 transphosphorylates STING at its C-terminal domains for IRF3 activation. The IRF3 then displaces to the nucleus and induces immune-stimulated genes and type I IFN expression. The nuclear factor kappa-light-chain-enhancer of activated B cells (NF-κB) signaling can also be activated by STING. Abbreviations: cGAMP, 2′,3′-cyclic GMP-AMP; ER, endoplasmic reticulum; IRF3, interferon regulatory factor 3; NF-κB, nuclear factor kappa-light-chain-enhancer of activated B cells; TBK1, TANK-binding kinase 1

The upstream dsDNA interacts with enzyme cGAS in a sequence-independent way [12, 13], promoting a conformational change of cGAS to catalyze the formation of 2′,3′-cyclic GMP-AMP (cGAMP), a cyclic dinucleotide (CDN) from ATP and GTP, containing the phosphodiester linkages of both 2′–5′ and 3′–5′ [14]. The cGAS activation as well as cGAMP synthase activate protein STING, in which the STING undergoes endoplasmic reticulum (ER)-to-Golgi trafficking and tetramer formation via a higher-order oligomerization [15] (Fig. 2). Palmitoylation of STING in Golgi is proposed for TANK binding kinase 1 (TBK1) as well as interferon regulatory factor 3 (IRF3) recruitment. The STING tetramerization induces recruitment and activation of TBK1 dimers, and TBK1 transphosphorylate STING at its C-terminal domains for IRF3 activation [16]. The IRF3 then displaces to the nucleus and induces immune-stimulated genes (ISG) and type I IFN expression [13]. The NF-κB signaling can also be activated by STING (Fig. 2).

### Biology and expression of protein STING and its pathway

The structure of protein human STING (hSTING) comprises a N-terminal trans-membrane domain with four helices (aa 1-154), an acidic C-terminal tail (aa 342-379), and a central globular domain (aa 155-341) separated the former two [17]. The mouse STING (mSTING) presents 81% similarity and 68% amino acid identity with hSTING, and the different sequence alleles were reported [18]. STING is present variously in different tissues, and its expressions in the skeletal muscle, brain, kidney, small intestine, colon, and liver were poorly found [4].

STING-deficiency has been reported to correlate with cancer incidence. In six cancerous melanoma cell lines (G361, MeWo, SK-MEL-5, SK-MEL-2, SK-MEL-28, and WM115), STING expression was not detectable or significantly inhibited [19]. Several colorectal adenocarcinoma human cell lines have described low or defective STING pathway activity, which was correlated with poorer Dukes’ stage [20]. Also, the STING silencing was observed in *KRAS*-mutated lung cancer, with the loss of the tumor suppressor gene *LKB1* [21]. Further studies of a co-culture of tumor-immune cells revealed that a downregulated cGAS-STING pathway could induce cancer resistance to immune effectors [22]. Their study also showed the relationship between the decreased intratumoral CD8^+^ T cell infiltration and downregulated cGAS-STING pathway mediated via the reduction of the expressions of the downstream IFN-I targeted genes such as chemokine (C-X-C motif) ligands 10 (*CXCL10*) [22]. Surviving cancer cells tend to harbor deficiencies in the cGAS-STING pathway under selective pressure [23].

### cGAS-STING pathway in cancer-immunity cycle

**Fig. 3:**
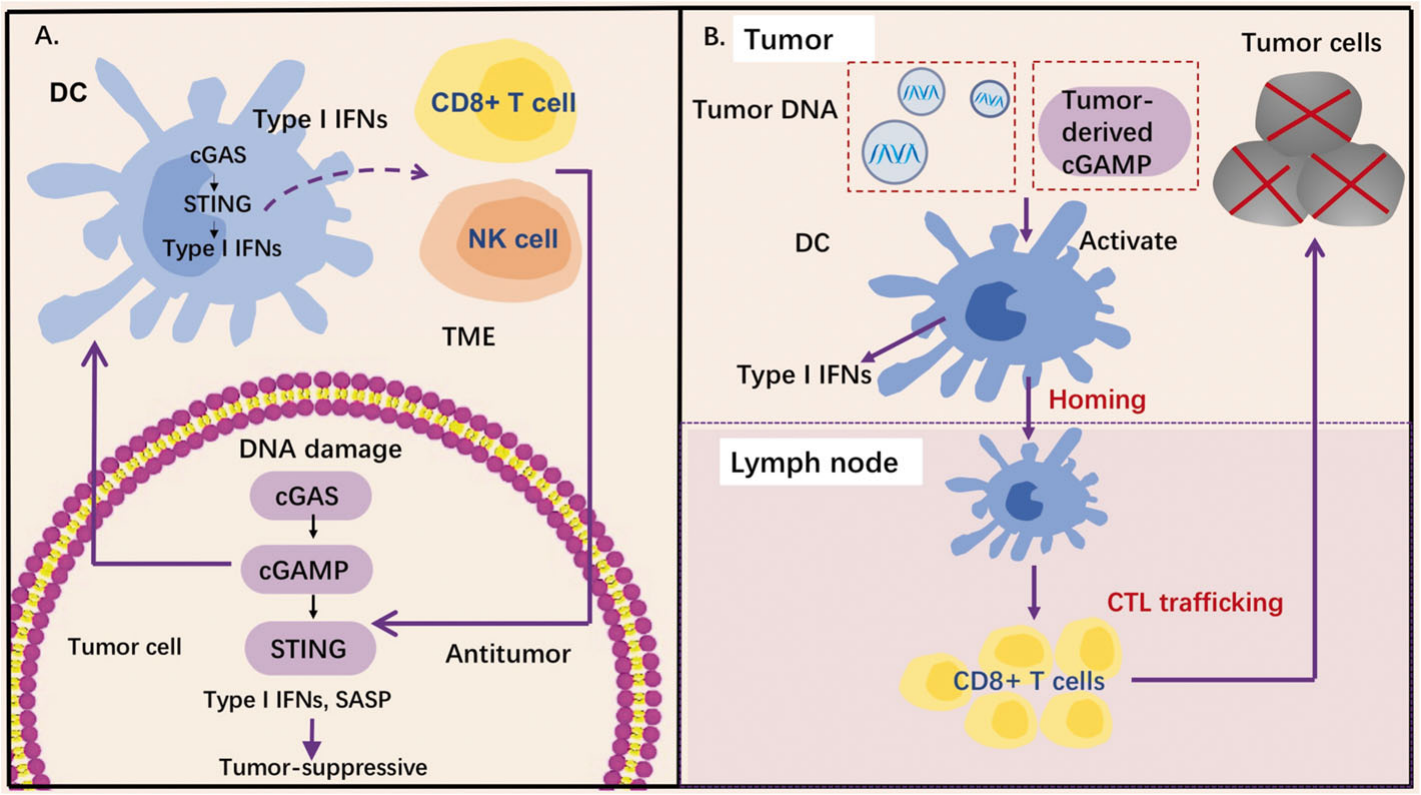
Role of STING pathway in tumor suppression. **a** cGAS-STING pathway and cancer-immunity cycle. cGAS-STING pathway functions as a tumor suppressor induced by DNA damage. Cytosolic DNA generated from different sources of DNA damage could be sensed by enzyme cGAS in a tumor cell. The cGAS then activates STING to upregulate type I IFN expression, which mediates tumor-suppressive effects. In addition, the cGAS-STING signaling allows the crosstalk between the tumor cells and immune cells nearby. Tumor-derived cGAMP or tumor-derived DNA could activate the activation of DCs, which activates the cGAS-STING pathway and promote immune cells against tumors. **b** cGAS-STING in innate immune sensing and spontaneous anti-tumor T cell responses. Tumor-derived DNA can induce cGAS-STING pathway activation of APCs and upregulate expression of type I IFNs, which increases its lymph node-homing capability and spontaneous T cells. Abbreviations: cGAMP, 2′,3′-cyclic GMP-AMP; CTL, cytotoxic T lymphocytes; IFN, interferon; NK cells, natural killer cells; SASP, senescence-associated secretory phenotype; TME, tumor microenvironment

The activation of the cGAS-STING pathway plays a crucial role in both tumor cells and immune cells as an innate immune sensor, which could regulate multiple steps in cancer-immunity cycle. This cytosolic DNA sensing has been well-characterized, which can induce IFN production and arouse host immune responses mediated by infiltration of immune cells such as T cells and natural killer (NK) cells [24, 25]. Activation of the cGAS-STING pathway in tumor cells may pose an obstacle to the progression of early neoplastic cells by upregulating type I IFNs or other inflammatory genes (Fig. 3a). Importantly, the cGAS-STING pathway has also been robustly linked to the induction of cancer cell senescence [26], thereby mediating the oncosuppressive effects. The capability of cGAS-STING signaling to promote senescence is dependent on the secretion of the chemokines, pro-inflammatory cytokines, growth factors, and proteases, which are components of the senescence-associated secretory phenotype (SASP) [26–28]. These immune-stimulatory factors can either contribute to the tumor control in a tumor-cell autonomous manner or arouse immune cells against tumors [26, 29, 30].

Pre-clinical studies have demonstrated the activation of STING pathway in host immune cells is vital for IFN-β production [31], and in turn, the STING pathway and type I IFN signaling are revealed necessary for productive CD8^+^ T cell cross-priming via Batf3-lineage dendritic cells (DCs) [32] (Fig. 3b). Two major hypotheses have been prompted for the DC activation by cancer cells: tumor-derived DNA activates the DCs [31], or tumor-derived cGAMP directly activates the STING pathway via protein STING [25], thereby leading to the production of type I IFNs. The type I IFN signaling is important for CD8α^+^ DC survival and antigen retention, which enhances DC’s cross-presentation [33]. Besides, type I IFNs have been reported capable of upregulating the expression of CCR7, MIP-3beta, and Th-1 chemokines, which reinforces the capability of lymph node-homing [34]. Through gene expression profiling studies of tumor biopsies, type I IFN signaling was found correlated with adaptive T cell responses against tumor-specific antigens [35, 36]. In addition, basic experiments in type I IFNR−/− mouse models or mice with the absence of the downstream transcription factor Stat1, suggested significantly reduced tumor antigen specific T cell responses in vivo [36, 37]. Further studies will be needed to unveil how the tumor and host-immune cGAS-STING signaling cooperates to promote tumor suppression.

## The pharmacological effect of STING agonists as antitumor therapy

### STING-nucleotidic agonists

Due to the correlation between deficiencies in cGAS-STING pathway and surviving cancer cells, as well as the importance of cGAS-STING in the regulation of cancer-immunity cycle, STING agonists were developed to mimic this activation to enhance anti-cancer effects (Table 1). CDNs have been verified as the mediators of cGAS-STING pathway in the immune system. Their antitumor modulation was discovered first in c[di-GMP], in which the tumor progression of human colon cancer cells was inhibited [44]. The endogenous cGAS production 2′,3′-cGAMP showed reduced tumor size and prolonged survival in mice bearing colon adenocarcinoma CT26 tumors [45]. Intratumoral injection of 2′,3′-cGAMP in the B16F10 mouse model also significantly delayed tumor growth and reduced lung metastases [40]. The results of their experiments in the mouse melanoma model demonstrated that intratumoral injection of cGAMP enforced anti-cancer CD8^+^ T cell responses, and this ability could be further enhanced when both PD-1 and CTLA-4 were blocked. Their further studies showed this immune response depended on the production of type I IFNs from endothelial cells in TME, indicating the strategy of targeting tumor endothelial cells for melanoma immunotherapy. Another study enhanced STING activation of the tumor and lymph nodes by using the cGAMP-based nanoparticles, which enhanced the cytosolic delivery of cGAMP by promoting its endosomal escape and triggered the formation of a “hot” type TME with enriched T cell infiltration [46]. This novel therapy presented marked efficacy combined with ICIs. Further, the STING-activating nanoparticles also showed the potential to induce immunological memory against cancers. Indeed, these cured mice in the rechallenge experiments rejected the tumors in the contralateral flank [46]. Theses therapeutic benefits highlight the importance of STING signaling in anti-cancer immunity in tumors.

**Table 1:** The anti-tumor cGAS-STING agonists

Molecule	Type	Administration method	Development stage	Ref/note
c(di-GMP)	Prokaryotic CDNs	IT	Pre-clinical	[38]
3′,3′-cGAMP	Prokaryotic CDNs	IP	Pre-clinical	[39]
2′,3′-cGAMP	Eukaryotic CDNs	IT	Pre-clinical	[40]
ML-RR-S2-cGAMP	Synthetic CDN agonists	IT	Pre-clinical	[41]
ADU-S100	Synthetic CDN agonists	IT	Phase 1, Phase 2	[41]
ML-RR-S2-CDG	Synthetic CDN agonists	IT	Pre-clinical	[41]
DMXAA	Non-CDN agonists	IT	Phase1, Phase 2, Phase3	[42]
Amidobenzimidazoles	Non-CDN agonists	IV	Pre-clinical	[43]
ExoSTING	Novel STING agonists	IT	Pre-clinical	SITC 2018 P618
MV-626	ENPP1 inhibitor	IP	Pre-clinical	SITC 2018 P410
SB11285	Novel STING agonists	IP, IT, IV	Phase 1	AACR 2017 P-A25
STACT-TREX1	Novel STING agonists	IT, IV	Pre-clinical	SITC 2018 P235
SYN-STING	Novel STING agonists	IT	Pre-clinical	SITC 2018 P624

*Abbreviations*: *IT* intratumoral, *IV* intravenous, *IP* intraperitoneal, *SITC 2018* Society for the Immunotherapy of Cancer 2018 Annual Meeting, *AACR 2017* American Association for Cancer Research 2017 Conference on Tumor Immunology and Immunotherapy

Beyond naturally derived CDNs, synthetic CDNs with better properties were developed. The anti-tumor compound dithio CDN (ML RR-S2 CDN, also known as ADU-S100 or MIW815) showed a high binding affinity to hSTING alleles [41]. This CDN analog showed marked antitumor efficacy in various cancer mouse models, which made it become the first STING agonist entering clinical trials in advanced metastatic solid tumors or lymphomas, with the first results reported in 2018, at the Society for ImmunoTherapy of Cancer meeting [47]. The inclusion criteria included 18-years old or older patients with advanced/metastasis solid tumors or lymphomas, Eastern Cooperative Oncology Group performance status of 0-1, and two or more cutaneous or subcutaneous neoplastic lesions accessible for biopsy, with one that could be injected. This phase I study enrolled 41 patients heavily pretreated before: 3 (7.3%) patients had received at least one prior-line treatment, 34 (82.9%) patients had received at least two prior treatments, and 22 (53.7%) had exposed to the ICIs therapy prior. During treatment, 35 of them discontinued because of disease progression (*n* = 26), physician or patient decision (*n* = 8), and death (*n* = 1) [47]. Dose-limiting toxicities were not reported, and the common adverse events were mainly including pyrexia, pain at the injection site, and headache. Based on Evaluation Criteria in Solid Tumors, partial response was observed in two patients (Merkel cell carcinoma, anti-PD-1 antibody-naïve; parotid gland adenocarcinoma, PD1 antibody-refractory). Treatment continued for more than 6 months in four patients. Follow-up clinical trials of combinations of this compound with ICIs are ongoing, which will be presented in Table 2.

**Table 2:** STING agonists in clinical trials

Agent	Target	Cancer type	Phase	Clinicaltrial ID
ADU-S100(MIW815)	STING	Head and neck cancer	Phase 2	NCT03937141
ADU-S100(MIW815)+/− Ipilimumab	STING+/− CTLA-4	Solid tumors/lymphomas	Phase 1	NCT02675439
ADU-S100(MIW815) + PDR001	STING+PD-1	Solid tumors/lymphomas	Phase 1	NCT03172936
E7766	STING	Urinary bladder neoplasms	Phase 1	NCT04109092
E7766	STING	Lymphoma/advanced solid tumors	Phase 1	NCT04144140
GSK3745417	STING	Neoplasms	Phase 1	NCT03843359
MK-1454	STING	Solid tumors/lymphomas	Phase 1	NCT03010176
MK-1454 + pembrolizumab	STING+PD-1	Head and neck squamous cell carcinoma	Phase 2	NCT04220866
BMS-986301	STING	Solid cancers	Phase 1	NCT03956680
SB 11285	STING	Solid tumor	Phase 1	NCT04096638

### STING-non-CDN agonists

A growing body of evidence showed the pharmacological function of non-CDN agents in cGAS-STING activation. The initiated agonist targeting the STING pathway is dimethyloxoxanthenyl acetic acid (DMXAA) [48]. Actually, DMXAA was first used as an anti-angiogenesis drug. However, the treatment of DMXAA failed the phase III trials in non-small cell lung cancer patients with no significant benefit brought [49]. The fact is that DXMAA is actually a competitive mSTING agonist with strong affinity, but not for hSTING [50]. Conlon and colleagues [50] found DMXAA and STING interacted restrictedly in mice, but too poor in human to promote type I IFN production.

The design of agent amidobenzimidazole (ABZI) represented a new breakthrough of STING agonist in immune-modifying cancer treatment [43]. This novel STING agonist was reported with significantly enhanced binding affinity using the 4-carbon butane linker (di-ABZI) for dimerization. The evaluation of STING activity was identified by IFN-β, and di-ABZI showed lower EC50 concentration than cGAMP. Treatment of di-ABZI in mice with subcutaneous CT-26 tumor-induced tumor regression and survival increase, and specially, 80% treated animals remained tumor-free until the end of this study. To our knowledge, this molecular is the initiated non-CDN agonist with competitive antitumor efficacy and hSTING selectivity.

## Applications of STING pathway in cancer immunotherapy

### STING agonist as a cancer vaccine adjuvant

Appropriate adjuvants play an essential role in tolerance overcome and tumor-specific immunity enhancement, and innate immunity activation is able to boost antigen-presenting cell (APC) activation, which facilities the immunogenicity of tumor-associated antigens (TAAs) [51]. STINGVAX is regarded as the first designed STING-based cancer vaccine, containing both the cancer cells secreting granulocyte-macrophage colony-stimulating factor (GM-CSF) and CDNs [52]. The STINGVAX injection in the contralateral part of the B16 transplanted melanoma, significantly inhibited the tumor size with a dose-dependent effect. The combined STINGVAX enhanced T cell infiltration in tumor tissues compared with the vaccine of single GM-CSF-secreting cancer cells. Besides, several tumor-bearing mice models demonstrated the strong antitumor effect of STINGVAX. Feasibility of STING-based cancer vaccine was verified later in mice bearing pancreatic cancer and melanoma [53, 54]. In addition, recently, Miao et al. identified and designed an effective STING-dependent cyclic lipid nanoparticles (LNP), as the adjuvant of antigen-specific mRNA vaccine delivery [55]. This research team developed multiple synthetical lipid structures using a one-step three-component reaction method, in which the lipids with a cyclic amino head group could activate the STING pathway. In the mouse model, the application of this combinatorial LNP demonstrated marked survival advantage, which showed a promising role of STING agonists in antitumor therapy [55].

### STING agonist combined with ICIs

Several STING agonists have been used as an anti-cancer therapy in clinical trials, and the STING agonists/ICIs combinations were also developed (Table 2). cGAS-STING agonists are ideal partners for ICIs. Firstly, the STING signaling and type I IFNs play crucial roles in spontaneous T cell responses via the cross-present of CD8_α_^+^ DCs [37], which promotes the intratumoral T cell infiltration. Importantly, high densities of adaptive immune cells (CD3^+^, CD8^+^, GZMB^+^, and CD45RO^+^ cells) represent favorable prognosis and positive clinical results for cancer patients [56]. Cytotoxic lymphocyte (CTL) infiltration is also regarded as an indicator for optimal response to ICIs. Besides, cGAS-STING pathway agonists can also increase antigen-presenting molecules such as Tap1, Tap2, and MHC-I with IFN upregulation, which may enhance the tumor immune surveillance [57]. In addition, STING agonists can increase tumor cells’ sensitivity to immune NK cells and CTLs [22]. Indeed, NLRX1 and NLRC3 proteins that could downregulate STING-mediated IFN-I signaling were increased in resistant tumor cells [22].

#### STING agonist combined with anti-CTLA-4 immunotherapy

The threshold of T lymphocyte activation can be reduced by the application of anti-CTLA-4 therapy [58]. Evidence suggests that the integrity of the STING pathway is essential to the optimal effect of CTLA-4-based immunotherapy [59]. Under the treatment of ionizing radiation combined with an-CTLA-4 therapy, Shane’s group found that STING absence prevented abscopal tumor regression, and deficient STING significantly impaired CD8+ T cell infiltration in the tumor tissues. Ager’s group also made a relevant study [38]. Their result showed the administration of combined anti-CTLA-4 therapy (9H10), anti-PD-1 therapy (RMP114), and agonistic anti-4-1-BB therapy (3H3), induced bilateral tumor regression in 40% of mice while the STING agonist CDG added, markedly inhibited the bilateral tumors in 75% of these mice. Therefore, the combination of CDG and ICIs effectively enhanced the antitumor effect.

#### STING agonist combined with anti-PD-1/programmed death-ligand 1 (PD-L1) immunotherapy

The cGAMP/antigenic peptide nanosatellite vaccine SatVax, combined with anti-PD-L1 therapy in the xenograft model, showed elevated E7-specific CD8+ CTLs, and decreased ratio of CD8+ Tim3+ and CD8+ PD-1+ T cells [22]. This successful combination led to significant tumor control, with four completely tumor-free mice of five animals. Reduced and delayed tumor growth was also showed in the B16 melanoma mouse model, treated by the co-administration of CDN-based poly beta-amino ester (PBAE-CDN) nanoparticles and anti-PD-1 therapy [60]. In addition, this combined therapy provided the mice with protection to tumor rechallenge [52]. Another advantage of this combined treatment is that the application of anti-PD-1/PD-L1 blockers can neutralize the STING agonist’s immunosuppressive effect. The upregulation of PD-L1 expression was reported in the cGAS-STING activation [57].

### STING pathway as a prognostic predictive biomarker in oncolytic immunotherapy

Oncolytic viruses have been regarded as a versatile platform to treat cancer. They are viral vectors that can kill tumor cells selectively and also have the potential to amplify the immune response and enhance anti-tumor effects [61]. The oncolytic immunotherapy talimogene laherparepvec has demonstrated a therapeutic benefit in patients with advanced melanoma in a phase III clinical trial [62]. The integrity of the cGAS-STING pathway is critical for response to the invasion from multiple pathogens and tumors, while cancers including melanoma and colon cancers are common with its deficiency [19, 20], as mentioned above. Based on these findings, it is reasonable to select the oncolytic immunotherapy to treat STING-loss cancers. In the melanoma mouse model, the herpes simplex virus type 1 (HSV-1) with γ34.5 gene deficiency was used to test its effect on cancer [19]. Under normal conditions, the existence of HSV-1Δγ34.5 could activate the STING signaling pathway effectively and help the host to clear its infection. Interestingly, in their study, the STING-deficiency melanoma cells were observed susceptibility to the virus infection while cancer cells with intact STING pathway grew rapidly. Additionally, a similar observation was also found in STING-loss mice with ovarian cancer [63]. Given that the STING deficiency correlates with an improved prognosis with oncolytic virus treatment, with further in vivo and clinical trials, it may represent a prognostic/predictive biomarker for oncolytic immunotherapy in cancer patients.

### STING agonist combined with CAR-T therapy

Engineered T cell has the ability to recognize the targeted antigen of tumor cells with the single-chain variable fragment domain, through transferring gene encoding chimeric antigen receptor (CAR) [64]. The CAR-T therapy is successful in several hematological diseases, but its application in solid tumors is limited [65]. The immune killing of CAR-T cells can be escaped mainly due to immunosuppressive TME and tumor heterogeneity [66, 67]. A new implantable bioactive device has been tested its property to deliver the CAR-modified T cells to the surfaces of tumors [68]. Although the delivery of this novel carrier promoted T cell expansion and a temporary tumor regression, antigen-negative tumors could not be eliminated completely. They further found that ribonucleic acid export 1 (RAE1)-high tumor cells were destroyed and the cells with RAE1 loss/low expression still survived. Thus, cyclic di-GMP (cdGMP), a STING agonist, was applied. Combination therapy of cdGMP and CAR-T cells led to a significant activation of the host APCs and lymphocyte responses, which eradicated tumors completely in four of ten pancreatic-bearing mice, with longer survival [68]. Meanwhile, after re-injection of tumor cells, these four tumor-free mice inhibited tumor growth to measurable mass. This CAR-T/cdGMP presented durable antitumor ability. The mechanisms how the corelease of STING agonists and CAR-T cells activates the host immunity remain to be fully elucidated.

## The challenge of STING-targeted immunotherapy against cancer:an emerging pro-tumor role of cGAS-STING

**Fig. 4:**
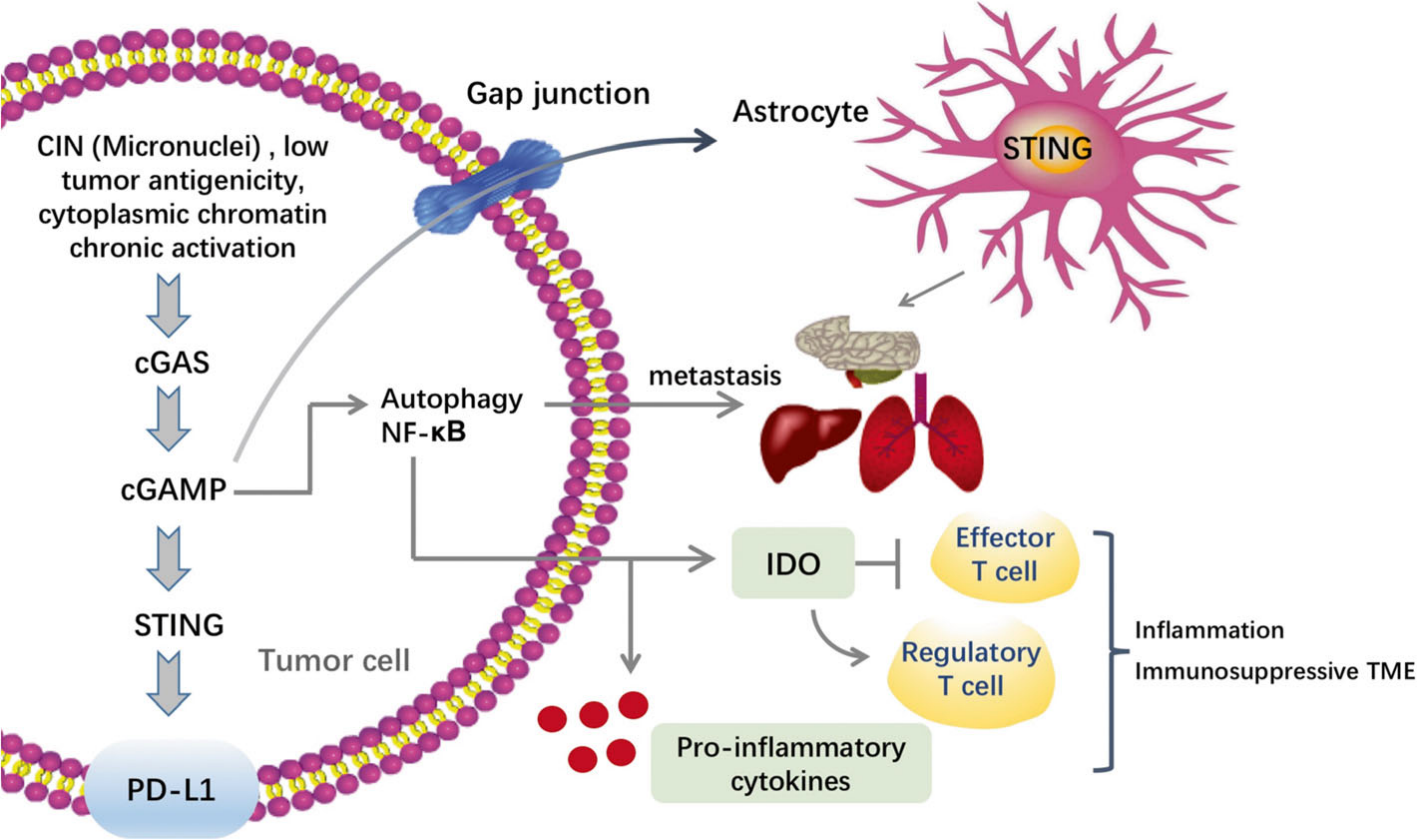
Regulation of cGAS-STING pathway in tumor promotion. The cGAS-STING pathway could exert its pro-tumor role in metastatic tumor settings. STING functions as a platform for different tumorigenic programs. High chromosome instability (CIN) tumors generated micronuclei, and its rupture could release DNA to the cytosol, enhancing the sense of cGAS-STING. Low tumor antigenicity and cytoplasmic chromatin chronic activation contribute to malignancy formation, through the activation of the cGAS-STING pathway. Chronic activation could also downregulate the expression of type I IFNs, upregulate noncanonical NF-κB signaling, and promote tumor metastasis. Indoleamine 2,3-dioxygenase (IDO) and anti-inflammatory cytokines released from the tumor induces the formation of an immunosuppressive TME. STING may also promote immune evasion and tumor metastasis by PD-L1 upregulation and autophagy induction. In addition, cGAMP, particularly, was reported to transfer from the tumor cells through gap junctions to astrocytes, promoting NF-κB and IFN signaling and inducing brain metastasis ultimately. Abbreviations: cGAMP, 2′,3′-cyclic GMP-AMP; IDO, indoleamine 2,3-dioxygenase; PD-L1, programmed death-ligand 1; TME, tumor microenvironment

Undoubtedly, STING agonists showed impressive potential in antitumor immunity. However, emerging evidence suggested the pro-tumor roles of the cGAS-STING pathway, from tumor initiation and development, to metastasis [69–72] (Fig. 4), which makes the application of STING agonists in the clinic remains a lot to challenge. First, different from acute STING-induced SASP, chronic SASP-correlated inflammation relates to malignant behaviors such as immune-suppression and oncogene-driven senescence evasion [26, 73]. Similarly, high chromosome instability (CIN) tumors generated micronuclei, and its rupture could release DNA to the cytosol, enhancing the sense of cGAS-STING. This regulation was reported related to the secretion of the pro-inflammatory cytokines by activation of NF-κB signaling and metastasis [74] (Fig. 4). When malignancies tolerate the long-term use of STING agonists, and lose the cell-cycle regulators downstream, inflammatory processes are able to function their pro-tumor effects. Apart from the intrinsic cGAS-STING activation in malignancies, metastasis could also be induced in a cancer cell non-autonomous manner. cGAMP, particularly, was reported to transfer from the tumor cells through gap junctions to astrocytes, promoting NF-κB and IFN signaling and inducing brain metastasis ultimately [69]. STING upregulation was also correlated with increased infiltration of regulatory T cells [70], and immune-regulatory enzyme indoleamine 2,3-dioxygenase (IDO), which can mediate tumor immune evasion and inhibit T cell proliferation [75]. Thus, chronic cGAS-STING activation may promote tumor metastasis which needs to be overcome. An important unanswered question existed is how these cancer cells change the STING’s downstream circuitry to mediate metastasis. One hypothesis supports that the precise control of levels of STING expression may be involved in this alteration [76]. In this study, the relationship between STING signaling magnitude and the apoptotic programs in T cells and macrophages was demonstrated. To overcome this challenge, further examinations are warranted to unveil the molecular requirements and regulations that function in metastatic promotion or suppression downstream of cGAS-STING cascade.

Beyond the pro-tumor chronic inflammation and CIN, the ER stress response as well as autophagy also serve as a barrier to the anti-tumor effect of STING agonists. They play a part in the advanced tumor progression by making tumor cells survive under stressful conditions [77]. The ER stress response enables disseminating tumor cells to exert the potentials of immune evasion [78]. cGAS-STING signaling was also revealed able to cooperate with the autophagy-ER stress responses to promote tumor progression [79] (Fig. 4). Additionally, metabolic programs may also be exploited by TME to induce immune suppression. The uptake of glucose or other nutrients in intratumoral T cells was restricted by tumor milieu, which results in ER stress and activation of the response of IRE1α–XBP1 unfolded protein. Chronic IRE1α–XBP1 activation makes T cells into a dysfunctional state, with reduced antitumor effects [77]. Further investigations are needed to discover the molecular hierarchy of ER stress and the cGAS-STING pathway, as well as how they interact to promote tumorigenesis.

Another major challenge is how to carefully select patients to enhance clinical response to STING agonists. The preliminary clinical phase 1 trial of STING agonist (MK-1454) in solid tumors and lymphomas has shown only a modest clinical response using single-agent treatment with no marked activity seen, and the co-administration of it with ICIs in advanced cancers showed only partial responses [80]. Therefore, novel STING agonist ABZI was developed by systemic administrated to patients [43]. Its safety remains to be considered, and hemodynamics of patients using such drugs need to be closely monitored. To determine which patients STING agonists might benefit is necessary. Different CIN state in primary and metastatic tumors indicates the active CIN might become a biomarker to predict personalized administrations. In fact, it is metastatic but not primary tumors, correlate with increased CIN, chronic STING activation, as well as poor patient prognosis [74]. Therefore, effective biomarkers and selection schemes are required to identify the patients who might benefit from STING agonists.

In addition, there may have technical limitations of the bulk RNA sequencing since the relationship between patient outcomes and cGAS-STING RNA levels is incongruent across different tumors. For example, poor patient survival was revealed with downregulated cGAS-STING signaling in a subset of human tumors [28, 72]. While in patients with colorectal cancer, high STING expression has also been demonstrated related to poor prognosis [81]. Contaminating stromal cells may mask the actual expression of cGAS-STING, or this difference reflects both pro-tumor and anti-tumor roles of cGA-STING in specific tumor types.

## Implications of the cGAS-STING pathway in cancer therapy

A comprehensive mechanistic understanding of the biologic STING signaling may greatly help to develop agents to potently activate it while reducing its immune-suppressive effects. For example, several feedback loops were found to attenuate STING activation including autophagy induction, AIM2 inflammasome activation, and autocrine IFN signaling [82, 83]. Compounds designed to impede one or more loops might greatly interact with the STING agonists. Additionally, a recent study demonstrated STIM1 anchors the STING to the ER membrane as a calcium sensor. In fact, deficiency of STIM1 can lead to spontaneous activation of STING with type I IFN production. This makes the STIM1-targeted inhibitor a potential strategy for future therapies [84]. Although the application of STING agonists is exciting, the recent correlation between the cGAS-STING pathway and metastasis also suggests the prospect for STING inhibition in late-stage cancers. Importantly, a recent covalent STING palmitoylation inhibitor has been discovered to attenuate metastasis in its therapeutic interventions [85]. Therefore, a personalized method of the use of STING agonists and antagonists may be helpful.

Another implication for selectively promoting desired outputs is to increase the local concentration of type I IFNs. One method is the tumor-targeted monoclonal antibodies combined with IFN-β. For example, a method of coupling either anti-EGFR or anti-Her2 monoclonal antibodies to IFN-β can result in tumor regression in tumor-bearing mice [86]. In addition, the type I IFNR^−/−^ mice lost the antitumor effect, indicating the importance of host immune cell priming [86]. Thus, transient expression of low IFN-β doses in TME may elevate tumor adaptive immune response.

It is also critical to understanding the STING-based TME changes in pre-clinical experiments and clinical trials, so that less side effects may be produced in cancer treatment. Most STING agonists have not encountered pro-tumor effects because just a few doses of treatment could result in a burst of type I IFN production to activate anti-tumor immune system [83]. Therefore, selecting an appropriate dose is vital. For instance, in mouse models, the ADU-S100 injection with low, single dose appears to produce effective tumor-associated T cell responses, while high repetitive doses may impair both T cell response and immune memory formation. Based on this, lower doses might be more helpful because of their ability to generate adaptive immune responses [87]. In addition, given the PD-L1 upregulation observed in STING activation, the combined therapy of STING agonists with anti-PD-1/PD-L1 antibodies would be extremely helpful for anti-cancer therapies.

## Conclusion remarks

The burgeoning interest in the STING pathway, using patients’ own immunity to eradicate tumors, is extremely appealing and several STING agonists were developed for cancer treatment with promising pre-clinical results. As a critical immune sensor, the STING pathway plays an important role in tumor control through tumor-derived DNA sensing and T cell priming. This induces T cell presentation in tumors, making the STING pathway a promising strategy combined with ICI therapy. STING agonists can elevate the efficacy of therapies from cancer vaccine, to ICIs to CAR-T immunotherapies. However, emerging pro-tumor roles have been described and a greater understanding of STING-associated TME and biologic mechanism is needed. We believe cGAS-STING pathway manipulation might become a promising strategy combined with cancer immunotherapy.

## Data Availability

Not applicable.

## Abbreviations

ABZ: Amidobenzimidazole
APC: Antigen-presenting cell
AACR 2017: American Association for Cancer Research 2017 Conference on Tumor Immunology and Immunotherapy
cGAS-STING: Cyclic GMP-AMP synthase-stimulator of interferon genes
CTLA-4: Cytotoxic T lymphocyte antigen 4
cGAMP: 2′,3′-cyclic GMP-AMP
CDN: Cyclic dinucleotide
CTL: Cytotoxic lymphocyte
CAR: Chimeric antigen receptor
cdGMP: Cyclic di-GMP
CIN: Chromosome instability
dsDNA: Double-stranded DNA
DMXAA: Dimethyloxoxanthenyl acetic acid
DCs: Dendritic cells
ER: Endoplasmic reticulum
FAA: Flavone 8-acetic acid
GM-CSF: Granulocyte-macrophage colony-stimulating factor
hSTING: Human STING
HSV-1: Herpes simplex virus type 1
ICIs: Immune checkpoint inhibitors
IFNs: Interferons
IRF3: Interferon regulatory factor 3
IDO: Indoleamine 2,3-dioxygenase
IT: Intratumoral
IV: Intravenous
IP: Intraperitoneal
LNP: Lipid nanoparticles
mtDNA: Mitochondrial DNA
mSTING: Mouse STING
NK cells: Natural killer cells
PD-1: Programmed death 1
PBAE-CDN: CDN-based poly beta-amino ester
RAE1: Ribonucleic acid export 1
SITC 2018: Society for the Immunotherapy of Cancer 2018 Annual Meeting
SASP: Senescence-associated secretory phenotype
TME: Tumor microenvironment
TBK1: TANK binding kinase 1
TAAs: Tumor-associated antigens
